# Peripheral B Cell Deficiency and Predisposition to Viral Infections: The Paradigm of Immune Deficiencies

**DOI:** 10.3389/fimmu.2021.731643

**Published:** 2021-08-30

**Authors:** Alexandros Grammatikos, Matthew Donati, Sarah L. Johnston, Mark M. Gompels

**Affiliations:** ^1^Department of Immunology, Southmead Hospital, North Bristol National Health Service (NHS) Trust, Bristol, United Kingdom; ^2^Severn Infection Sciences and Public Health England National Infection Service South West, Department of Virology, Southmead Hospital, North Bristol NHS Trust, Bristol, United Kingdom

**Keywords:** B cells, alymphocytosis, viral infections, immunodeficiency, immune deficiency, humoral immunity, immune responses

## Abstract

In the era of COVID-19, understanding how our immune system responds to viral infections is more pertinent than ever. Immunodeficiencies with very low or absent B cells offer a valuable model to study the role of humoral immunity against these types of infection. This review looks at the available evidence on viral infections in patients with B cell alymphocytosis, in particular those with X-linked agammaglobulinemia (XLA), Good’s syndrome, post monoclonal-antibody therapy and certain patients with Common Variable Immune Deficiency (CVID). Viral infections are not as infrequent as previously thought in these conditions and individuals with very low circulating B cells seem to be predisposed to an adverse outcome. Particularly in the case of SARS-CoV2 infection, mounting evidence suggests that peripheral B cell alymphocytosis is linked to a poor prognosis.

## Introduction

Humoral immunity has been regarded as the pillar of immune defenses against bacterial infections, but was thought to be less essential for viral infections. For the latter, cellular immunity was traditionally considered to be more important, e.g. T and Natural Killer (NK) cells. Data from the world of primary immunodeficiency seems to support this view: patients with Common Variable Immune Deficiency (CVID) generally present with recurrent bacterial infections, while those with Severe Combined Immune Deficiency (SCID) are also predisposed to viral infections (1, 2).

This is however only a simplified paradigm and in reality immune defenses against infection depend on more complex and interlinked processes for example, antibodies are known to be important in neutralizing viral particles and preventing them from entering human tissues (3). They also participate in the elimination of virally infected cells, *via* the process of opsonization and subsequent phagocytosis (3). IgA is known to play a vital role in antiviral defenses at mucosal sites, e.g. respiratory or gastrointestinal (4).

The importance of humoral immunity in the protection from viral infection is most elegantly exemplified by immunization, e.g. to measles, chicken pox and rubella viruses. Protective immunity by these vaccines relies largely on the development of an adequate humoral immune response to the virus and serology levels are often used as a proxy to assess immunity (5). Viral vaccines that depend solely on T cell-mediated immunity on the other hand are yet to be implemented successfully in clinical practice (6). Another paradigm is the placental transfer of protective IgG antibodies from mother to the fetus, which confers protection from some viral infections during the first months of life (7).

In our attempt to understand the role of the various parts of the immune system in the defense against infections, immunodeficiencies present a valuable model. Immunodeficiencies with absent or very low B cells in particular are very useful to understand the role of these cells in antiviral immunity. The best example is offered by patients with agammaglobulinemia (X-linked or Autosomal Recessive), but patients with Good’s syndrome, those that have undergone B cell-depleting therapy, and certain patients with CVID are also very informative in this respect.

To identify relevant articles in the literature we searched PubMed and Google scholar databases using the following terms: immunodeficiency, immune deficiency, viral disease, viral infection, peripheral B cells, serum B cells, B cell lymphopenia, B cell alymphocytosis, X-linked agammaglobulinemia, XLA, autosomal recessive agammaglobulinemia, Good’s syndrome, common variable immune deficiency, CVID, rituximab, B cell depleting therapy, anti-CD20 monoclonal-antibody therapy, SARS-CoV-2, COVID-19, enteroviruses, JC virus, astrovirus, adenovirus, measles, mumps, rubella, BK virus, West Nile virus, norovirus, HSV, CMV, VZV, HHV7, HHV8, HPV, vaccinia virus, parvovirus B19 and hepatitis virus. Reference lists of retrieved articles were also manually screened for relevant studies.

## XLA and AR Agammaglobulinemia

X-linked agammaglobulinemia (XLA) manifests with markedly reduced peripheral B cells, severe hypogammaglobulinaemia and recurrent sinopulmonary infections. The latter are largely bacterial but the role of viruses is recently starting to be recognized (**Table 1**). Several cases of atypical, unusually severe, recurrent or persistent viral pneumonia have been described in these patients (18, 62, 147, 148). More recently, many cases of persistent SARS-CoV2 pneumonia have been reported, highlighting the importance of B cells in the defense against this virus (132–136).

**Table 1:** Viral infections in patients with agammaglobulinemia, Good’s syndrome, CVID and post-B cell-depleting therapy.

	Agammaglobulinemia (XL or AR)	CVID	Good’s syndrome	Post anti-CD20 therapy
**Pathogenesis**	Mutations in genes linked to B cell maturation	Various/unknown	Unknown	Iatrogenic
**Circulating B cells**	Very low or absent	Low in 10% of cases	Low or absent	Low or absent
**Viral infections**				
Enteroviruses (Echovirus, Coxsackie virus and Poliovirus)	Chronic meningoencephalitis, dermatomyositis-like syndrome, chronic diarrhea, arthritis, post-vaccination poliomyelitis (8–25)	Chronic meningoencephalitis, post**-**vaccination poliomyelitis, asymptomatic infection (19, 20, 26–31)	Meningoencephalitis^*^	Chronic meningoencephalitis, dermatomyositis-like syndrome, acute hepatitis (32–51)
JC virus	Progressive multifocal encephalopathy (19)	Progressive multifocal encephalopathy (29, 52)	Progressive multifocal encephalopathy (53–56)	Progressive multifocal encephalopathy (57, 58)
Astrovirus	Encephalitis (59, 60)			
Adenovirus	Dermatomyositis-like syndrome, arthritis (11, 61)	Chronic lower respiratory tract infection (62), meningoencephalitis (27)		Fulminant hepatitis (63)
Measles		Vaccine**-**associated inclusion-body encephalitis (64)		Fatal pneumonitis (65)
Mumps		Encephalitis (29)		
Rubella		Cutaneous and visceral granulomas (66–69)		
BK virus		Encephalitis (70)		Nephropathy, viremia (71)
West Nile virus		Meningitis (72)		Neuroinvasive disease (73–77)
Norovirus	Chronic diarrhea/fecal shedding (24)	Chronic diarrhea (78–82)		Chronic diarrhea (83)
HSV	Severe oropharyngeal infection (84)	Encephalitis (85, 86)	Ulcerative dermatitis, recurrent genital, esophagitis, epiglottitis, acute hepatitis, keratitis. meningitis (87–93)	Encephalitis (94), keratitis (95), tracheitis (96)
CMV		Chronic diarrhea (97), retinitis (98) and pneumonia (22)	Retinitis, hepatitis, colitis, enteritis, gastritis, pneumonia, encephalitis (87, 90, 91, 99–106)	Meningoencephalitis (107, 108), pneumonitis (109, 110), colitis (111)
VZV	Chickenpox (112)	Angiitis (113)	Disseminated or cutaneous infections (87, 88, 114)	Disseminated (including post vaccination) or cutaneous infection (33, 34, 71, 115, 116)
HHV7	Severe liver disease (117)			
HHV8		Interstitial lung disease and lymphoproliferative disorder (hypothesized) (118)	Kaposi’s sarcoma (119)	
HPV		Severe warts (22, 120, 121)	Severe warts (122)	Severe warts (123)
Vaccinia virus	Disseminated infection (11, 17, 124)			
Parvovirus B19		Arthritis (125)		Peripheral blood cytopenias (126–131)
SARS-CoV2	Persistent pneumonia (132–136)	Severe pneumonia (137–139)	Severe pneumonia (139)	Persistent/severe pneumonia (140–143)
Hepatitis B virus				Reactivation (34, 144–146)

^*^authors’ personal experience.

Enteroviral infections are also well documented in these patients; chronic meningoencephalitis being their most serious complication (8–16, 24). Mostly echoviruses, but also coxsackie and polio viruses have been reported (17, 18). Chronic enteroviral infection can also manifest as a dermatomyositis-like syndrome in some patients, with muscle weakness, rash and edema (8, 11). Cases of chronic diarrhea or arthritis from echoviruses have also been reported (11, 18, 61). Enteroviral infections are more common in untreated patients or those that are on suboptimal immunoglobulin replacement, again suggesting an important role for humoral immunity (19, 149).

Before it was recognized that live vaccines pose a significant threat in these patients, several cases of post-vaccine infection were reported, e.g. post-poliomyelitis vaccination (8) or disseminated vaccinia virus infection (11, 17, 124) (). In a recent systematic review, agammaglobulinemic patients exhibited the highest likelihood of developing vaccine-derived poliovirus infection in comparison to other primary immunodeficiency disorders (20). Interestingly, the risk was lowest in patients with SCID, suggesting that cellular immunity is less important for the protection against this virus (20).

Other viruses that have been linked to encephalitis in XLA include astrovirus (59, 60) and JC virus (19). Adenovirus has been linked to the development of a dermatomyositis-like syndrome and arthritis (11, 61). Finally, chronic fecal shedding of norovirus is very common in XLA children, occurring in 40% of cases (150). Interestingly, EBV and herpes-viral infections are only rarely reported in these patients (112, 117, 151).

AR mutations in the genes encoding for IgM heavy chain, Igα (CD79α), λ5, BLNK and LRRC8 cause a similar disease phenotype to XLA. These defects are very uncommon, but enteroviral infections have also been reported in these patients, both post-vaccination and naturally acquired (21–23, 25).

## Good’s Syndrome

Patients with Good’s syndrome present with thymoma and immunodeficiency. Hypogammaglobulinemia is most commonly described but very low or even absent B cells are also characteristic (90, 152). CD4 T cell lymphopenia is less frequent and seems to be less clinically relevant: T cell counts do not readily predict the risk of acquiring opportunistic infections in these patients, particularly cytomegalovirus (CMV) (90, 153). In the largest case series to date (n=78) the average level of peripheral CD4 T cells in patients with Good’s syndrome was normal, while B cells were virtually absent (152). The cause of B cell deficiency in these patients is unknown but certain evidence suggests that it is a bone marrow defect, with an underlying arrest in B cell development that may be responsible (153, 154). The clinical picture is similar to that of XLA, but opportunistic infections are more common.

Chronic or recurrent viral infections in particular are seen in up to 40% of patients, usually in the context of normal CD4 T cells (**Table 1**) (87, 90, 91). CMV infection is common, resulting in retinitis, hepatitis, colitis, enteritis, gastritis, pneumonia or encephalitis (87, 90, 91, 99–106). Other infections include chronic human papilloma virus (HPV; warts) (122), HSV (ulcerative dermatitis, recurrent genital infection, esophagitis, epiglottitis, acute hepatitis, keratitis or meningitis) (88-93), human herpesvirus 8 (HHV8; Kaposi’s sarcoma) (119), JC virus (progressive multifocal encephalopathy) (53–56) and VZV (herpes zoster or disseminated infection) (87, 88, 114). A case of severe SARS-CoV2 infection has also been documented in a patient with Good’s syndrome and absent B cells (139). Finally, a significant proportion of patients that developed yellow fever vaccine-associated viscerotropic disease post vaccination had a history of thymus disease, suggesting possible Good’s syndrome (155).

Although hypogammaglobulinemia is generally thought to be the main driver in this disease, B cell counts correlate better with the risk of infection (153, 156). Moreover, cases with normal immunoglobulins but absent B cells and recurrent infections are reported in the literature (157).Thymectomy can cure the autoimmune features of this disease, but not B cell lymphopenia, suggesting that this may be the primary defect in these patients (158).

## CVID

Patients with CVID generally have normal levels of circulating B cells, but in up to 10% of cases B cells can be low (159). Of note, it is patients with absent B cells that tend to have the worse prognosis (91).

Although bacterial sinopulmonary infections are typically documented in CVID, various atypical viral infections have been described and these may be more common than previously thought (**Table 1**). Kainulainen et al. found that latent or chronic lower respiratory tract viral infections are frequently detected in these patients, usually caused by adenovirus (62). Similar findings were noted in a more recent study of 1303 immunocompromised patients; viral infections were identified in 35% of 2666 bronchoalveolar lavage samples, with rhinovirus-enterovirus, parainfluenza and coronavirus being the most common (160). Severe SARS-CoV2 infection has also been documented in 2 CVID patients with very low peripheral B cells (137, 139). In a recent international study of patients with inborn errors of immunity, CVID patients comprised a large proportion of the total number of deaths from SARS-CoV2 (138).

Although less common in comparison to XLA, chronic meningoencephalitis by enteroviruses and post-vaccination poliomyelitis have also been described in these patients (19, 26, 27, 29). Asymptomatic infection is also common (30, 31). In a recent systematic review, CVID patients exhibited the lowest probability of clearing vaccine-derived poliovirus infection in comparison to other primary immunodeficiency patients (20). Serum B cell counts are unfortunately not generally cited in these cases, with the exception of a recent report of a patient with monogenic CVID and fatal enteroviral encephalitis (28). Peripheral B cells were absent in this patient and interestingly, her primary and secondary lymphoid tissues also lacked B cells (28). Throughout the initial course of her disease she was on adequate immunoglobulin replacement, suggesting that B cell lymphopenia may have been the primary driver of her poor clinical outcome (28).

Other reported viral causes of meningoencephalitis in CVID include BK virus (70), adenovirus (27), mumps (29), West Nile virus (72), and HSV (85, 86). Also JC virus-induced PML has been cited (29, 52). Measles inclusion-body encephalitis caused by the vaccine strain of the virus has been documented in a young boy with features suggestive of CVID (64).

Up to 10% of CVID patients develop an inflammatory bowel disease-like disorder, and viral infections are thought to play a key role in its pathogenesis (97). CMV has been implicated (97) but most commonly chronic norovirus infection is cited (78–82). Interestingly, these patients tend to have very low or absent peripheral B cells (78) and very few plasma cells in their intestine (161). Of note, even non-CVID patients that suffer from chronic norovirus infection tend to have absent B cells, suggesting a vital role for these cells against this virus (78).

Other severe or chronic viral infections that have been documented in CVID include VZV-induced angiitis (113), parvovirus B19-induced arthritis (125), and severe HPV-associated warts (22, 120, 121). Apart from enteritis, CMV infection has also been linked to retinitis (98) and pneumonia (22). Rubella virus vaccine strains are postulated to play a role in the development of the cutaneous and visceral granulomas that are seen in these patients (66–69, 162). Finally, HHV8 is suspected to play a role in the pathogenesis of interstitial lung disease and of lymphoproliferative disorders that are more frequent in these patients (118).

## B Cell-Depleting Therapies

Rituximab, an anti-CD20 monoclonal antibody, depletes peripheral B-cells and causes subsequent hypogammaglobulinemia with impaired specific-antibody production (163, 164). Its effects can last for several months, particularly when given in multiple cycles, and in many cases circulating B cells remain low long-term (165–168).

Rituximab has now been used in clinical practice for more than 20 years and its role as an independent risk-factor for the development of viral infections is well documented (34, 71). Cases of viral reactivation are reported in the literature (**Table 1**), most commonly by hepatitis B virus (34, 144–146), VZV (disseminated or cutaneous infection) (33, 34, 71, 115) and CMV (meningoencephalitis (107, 108), pneumonitis (109, 110), colitis (111)). JC virus-induced PML (57, 58), BK virus-associated nephropathy/viremia (71), severe warts (123), adenovirus-induced fulminant hepatitis (63), and HSV-induced encephalitis (94), keratitis (95) and tracheitis (96) have also been reported. These associations have led professional societies to recommend hepatitis screening before treatment initiation (169, 170) and/or herpes zoster prophylaxis post-treatment (171).

De-novo viral infections are equally common, with the most common examples being parvovirus B19-induced peripheral cytopenias (126–131) and West Nile virus neuroinvasive disease (73–77). More recently, several cases of persistent (140, 141, 143) or fatal (142) SARS- CoV2 infection have been documented in patients that previously had rituximab treatment. Several cases of enteroviral meningoencephalitis are also documented (32–45) suggesting that absent peripheral B cells, irrespective of the cause, is the key driver for this disease. Cases of enterovirus-induced acute liver failure are also cited (46, 47), as well as cases of chronic norovirus diarrhea (83) and fatal measles pneumonitis (65).A case of fatal, disseminated VZV infection following zoster vaccination was recently documented in a patient that previously had rituximab-inclusive chemotherapy (116). Some of the above studies included patients that were receiving additional immunosuppressive agents, making it difficult to delineate the individual contribution of rituximab to the patients’ immune status; but in several others, the role of rituximab as an independent risk factor was established (168, 172-174).

Not surprisingly, other anti-CD20 monoclonal antibodies have a similar risk-profile. Several cases of post-obinutuzumab enteroviral meningoencephalitis have been reported and these patients also sometimes develop a dermatomyositis-like syndrome (48–50). Finally, a patient with echovirus-induced fulminant hepatitis after ocrelizumab has been reported (51).

## Discussion

The COVID-19 pandemic has brought viral infections into the spotlight of global scientific interest and understanding how our immune system protects us from these infections is today more pertinent than ever. The immunodeficiency disorders discussed above offer an insight into the role of humoral immunity in antiviral defenses. Contrary to the common belief that patients with humoral immune defects are predisposed to bacterial infections alone, viral infections are frequently reported in these patients, particularly in those with absent peripheral B cells. Viral infections are of course also common in healthy individuals, who may also present with unusually severe or prolonged viral infections. The cases described above however highlight an atypical and opportunistic pattern of infections in patients with absent circulating B cells.

Chronic enteroviral infection is one of the most characteristic examples and strongly suggests that very low B cells are an important predisposing factor for an adverse outcome, irrespective of the cause. IgG replacement therapy helps prevent severe sequelae in most, but not all patients, suggesting that low circulating B cells may represent an independent risk factor. Persistent Norovirus infection is another example that is seen almost exclusively in individuals with low or absent circulating B cells, irrespective of the cause (XLA, CVID and even in the general population). B cells are also absent in intestinal biopsies from these patients, highlighting an important role for local humoral immunity. Mounting evidence also suggests that B cell alymphocytosis is linked to an adverse outcome from SARS CoV2 infection. In certain situations, cellular immunity seems to be even less relevant than humoral, e.g. XLA patients had a higher likelihood to acquire vaccine-derived poliovirus infection in comparison to patients with combined immune deficiencies.

Viral pathogenesis of course also plays an important role in the outcome of any viral infection. Most viruses spread freely in tissues *via* lytic cellular infection but some can also spread directly, from cell-to-cell. It is the latter types that are more protected by the effects of humoral immunity and lack of B cells would not be expected to make a significant difference to their survival. Indeed, herpes viruses are known to spread *via* direct cell-to-cell contact in neuronal networks (175) and this may explain why reports of severe or atypical herpes viral infections in adult XLA patients are lacking in the literature. Another example of a virus that is not reported frequently in patients with absent circulating B cells is Epstein Barr virus (EBV), in agreement with the notion that B lymphocytes likely represent the primary reservoir for this virus (151).

The examples discussed above are unlikely to be the only ones. Severe or persistent viral infections are much more likely to be diagnosed and/or be reported in the literature, but mild viral infections are likely to be frequent and may contribute to the development of other infections as well. In comparison to bacterial infections, viral infections tend to be more difficult to diagnose and widely available diagnostic tests are lacking for most of them. Another complicating factor is that serological methods tend to be unreliable in antibody deficient patients, both due to their dependency on native IgG production and due to the frequent external administration of therapeutic IgG. Furthermore, viral infections tend to be of lower clinical interest than bacterial, e.g. in lower respiratory tract infection, and are less often interrogated by clinicians. The small numbers of patients that suffer with congenital B cell alymphocytosis have made it more difficult for this association to be established in the past, but the more prevalent use of rituximab in recent years has helped bring it into focus. The few studies that have specifically looked into the frequency of viral infections in patients with agammaglobulinemia also lend support to this association.

There are several reasons why B cell alymphocytosis may confer a particular predisposition to viral infections. Peripheral B cells are generally a good marker of the overall content of tissue B cells and when absent this commonly translates into very poor antibody responses to infection. In most other humoral immunodeficiencies, e.g. in CVID patients with normal B cells, antibody responses may be sufficient enough to confer protection from viral infections. Patients with absent circulating B cells are very likely to have absent IgA and IgM antibodies, the latter having important roles in antiviral immunity; e.g. IgA in mucosal tissues and IgM as the first isotype responder to infection (176). Finally, B cells may also play an important role themselves, due to their ability to act as antigen-presenting cells and thus stimulate cellular immunity.

Of course, patients with defective humoral responses are less prone to serious viral infections than those with defective cellular immunity but that does not necessarily reflect a lack of importance of B cells in antiviral immunity. It rather highlights the importance of T helper cells in priming humoral immunity, *via* the generation of memory B cells and long-lived plasma cells. Humoral immunity is effective in neutralizing viral particles and thus preventing their spread through tissues but once an infection is established, cellular immunity is more important *via* its role in eliminating virally infected cells. In line with this notion, hyperimmune globulin, immunoglobulin preparations that are high in antibodies against a specific disease, are available for prophylactic use of several types of viral infections and can even prevent progression when used early on in the course of an infection (177–179). However, they tend to be less effective when it comes to treating an established viral infection (180, 181).

Understanding the importance of humoral immunity, and serum B cells in particular, in the defense against viral infections has important clinical implications. Firstly, circulating B cells may be used as a screening tool to risk-stratify patients that are infected by viruses. This may be particularly important for SARS-CoV2 infection, as is underlined by multitude of the recently published case reports. Secondly, physicians need to be vigilant with the use of B cell depleting therapies, particularly in those patients that have a weakened immune system. The need to monitor B cell counts in these patients has also been recommended in the past. Thirdly, strategies may need to be adapted to protect patients with very low serum B cells, e.g. through the use of infection control measures, antiviral prophylaxis and with prompt investigation and treatment of infectious episodes.

Clinicians caring for patients that harbor humoral immune defects will also need to be vigilant for viral infections and include them in their differential. Live viral vaccines need to be avoided in these patients. Previous cases of post-vaccination poliomyelitis have raised awareness and live OPV is now contraindicated in the above immunodeficiencies. Guidance on the use of other live vaccines is however less clear, e.g. the Green Book, a UK government publication, recommends that live vaccines should not be administered to individuals who are on or have received immunosuppressive biological therapy in the past 12 months (182). However, antibody deficiencies are not considered to be a contraindication (182). The Infectious Diseases Society of America on the other hand proposes that live vaccines should be avoided equally in patients with major antibody deficiencies (e.g. XLA or CVID) and in those on biologic immunosuppressive therapy (183). Similarly, the Immune Deficiency Foundation advises that antibody deficient patients with absent B cells should avoid live viral vaccines (184). The above literature adds support to this view and measuring circulating B cells may even offer an easy way to discriminate between CVID patients that can and those that should not receive these vaccines.

Overall, the evidence summarized above suggests that atypical and opportunistic viral infections are not uncommon in patients with low or absent peripheral B cells and offers us an insight into the importance of humoral immunity in the defense against viral infections. Physicians should be vigilant for these types of infections in patients with low B cells and follow appropriate prophylactic measures.

## Data Availability Statement

The original contributions presented in the study are included in the article/supplementary material. Further inquiries can be directed to the corresponding author.

## Author Contributions

AG: conceptualized, wrote first draft and edited manuscript. MD: edited manuscript. SJ: edited manuscript. MG: edited manuscript. All authors contributed to the article and approved the submitted version.

## Conflict of Interest

The authors declare that the research was conducted in the absence of any commercial or financial relationships that could be construed as a potential conflict of interest.

## Publisher’s Note

All claims expressed in this article are solely those of the authors and do not necessarily represent those of their affiliated organizations, or those of the publisher, the editors and the reviewers. Any product that may be evaluated in this article, or claim that may be made by its manufacturer, is not guaranteed or endorsed by the publisher.
